# An experiment on data sharing options designs for eHealth interventions

**DOI:** 10.1016/j.invent.2023.100642

**Published:** 2023-07-01

**Authors:** Valentina Bartali, Lex van Velsen

**Affiliations:** Roessingh Research and Development and University of Twente, Roessinghsbleekweg 33b, 7522 AH Enschede, the Netherlands

**Keywords:** User-centred design, eHealth intervention, Ease of use, Trust, Design for privacy

## Abstract

**Background:**

With eHealth technology interventions, users' personal health data can be easily shared among different stakeholders. Users should decide with whom they want to share their data. As support, most eHealth technology has data sharing options functionalities. However, there is little research on how to design these visually. In this paper, we took two possible data sharing options designs - data and party perspective – for an existing eHealth technology intervention, and we explored them.

**Objective:**

The aim was to find which of the two designs is the best in terms of trust, privacy concerns, ease of use, and information control. Additionally, to investigate how these factors influence each other with also the goal of giving practical advice on designing for privacy.

**Method:**

We conducted a between-subjects online design experiment (*N* = 123). After having visualised one of the two data sharing options designs, participants filled in an online questionnaire. To analyse the data, *t*-test analyses, correlation analyses, and backward regression analyses were conducted.

**Results:**

Information control scored higher in the data perspective condition (*t* (97) = 2.25, *p* = .03). From the different regression analyses, we found that trust and ease of use play a role in all sharing-related factors.

**Conclusions:**

We concluded that the design of data-sharing options in eHealth technology affects the experience of the user, mostly for trust and ease of use. In the end, we provided several actionable design advices on how to design for privacy.

## Highlights

• We tested two data sharing options designs in a developed eHealth intervention.
• We explored the designs via trust, ease of use, privacy, information control.
• Deciding with whom to share specific data can increase feelings of control.
• Data sharing options in eHealth need to have trustworthy design features.
• This paper gives some advises on how to design for privacy.

## 1 Background

The use of eHealth technological interventions for therapeutic purposes is rapidly increasing. This has many advantages for patients in managing their health and receiving treatment. However, often this also requires them to share their personal health data through technology. This personal data can be quite diverse, ranging from demographical data, to personal preferences, to health data. Most eHealth technologies are collaborative Health systems, which means that user's data are stored in one place and they can be shared with more than a person or institution (Kim et al., 2019), like a doctor, insurance company, or developers of the technology. This can be beneficial because, for instance, users can be monitored by their therapists from a distance or developers can use the data to improve the technology. However, not everyone may be willing to share all their personal data with some parties without being first informed or making conscious decisions; being in control is the right of the patient (Skär and Söderberg, 2018).

To ensure that users give informed consent on which data to share and with whom, some eHealth technologies have consent notices with data sharing options. However, it was found that the design of data sharing options can cause confusion if it is not according to users. Accordingly, in a study by Karampela et al. (2019) on user attitudes towards sharing medical personal data, it was recommended to technology developers to create user-friendly interfaces which can enable users to understand and choose which data they want to share with whom.

To do so, it is important to define several key concepts and see how these are connected. These are trust, privacy concerns, information control and ease of use. Within the context of eHealth services that make use of personal data, it is often difficult, if not impossible, for an end-user to understand what personal data is collected, and how this data is shared with external actors or organizations. Accordingly, the concept of *trust* plays an important role. Trust can be seen as “an individual's belief in the competence, dependability, and security of the [online health service] under conditions of risk.” (Kini and Choobineh, 1998, p. 51). In a situation where the end-users cannot judge how their data is dealt with, the decision whether or not to entrust an eHealth service with personal information is a matter of trust. The end-user forms an assessment of the trustworthiness of this service, based on different cues (e.g., interface aesthetics, statement of compliance with security norms), which fuels the decision to share data or not.

With collaborative Health systems, patients' information is stored in cloud data storage and it can be shared among different parties (Kim et al., 2019). Accordingly, some patients might feel like losing control over their personal data and with whom they are shared. This is linked to the definition of *information control*, which is about the degree of a person feeling in control of his or her own personal information (Taylor et al., 2009). When users feel that they do not have control over their own personal information because, for example, their information is shared, they might feel that their privacy is breached (Kim et al., 2015; Sahama et al., 2013, p. 250). In this case, it can be said that users might have *privacy concerns*: “concerns about possible loss of privacy as a result of information disclosure” (Xu et al., 2008, p. 4). Due to privacy concerns, users might not be willing to share their data (Abdelhamid et al., 2017). Moreover, privacy concerns might be seen as the opposite of trust as, according to the definition of trust, a system which gives users privacy concerns and the feeling of losing control might not be trusted.

Nonetheless, by sharing health information, patients could have better and more targeted therapy as each patient's physician can have easy and quick access to previous consultations (Pussewalage and Oleshchuk, 2016). Because of these benefits of data sharing options in eHealth technology interventions, these services should be developed by including privacy by design features, so to decrease the degree of privacy concerns and increase the degree of trust and information control. Privacy by design refers to including features which ensure privacy and perceived privacy in the design of a service (Cavoukian, 2009). Cavoukian (2009) explains seven foundation principles of privacy by design. However, of these seven, only one can be applied to the visual design of an eHealth service. This is the *Respect for User privacy* principle, which is about designing a service which is user-centric to keep the interests of the individual uppermost. To do that, users should be always asked for consent to collect, use or disclose personal data. Additionally, users should always have access to their data and change it as they please.

Following the same line of thought, Jensen and Potts (2007) presented the *Structured Analysis of Privacy (STARP)* framework, which is a user-centred privacy-aware design tool which helps to spot privacy vulnerabilities. This framework gives principles on how to visually design data sharing options which prompt awareness, ensure users have clear choices, ensure the integrity and security of data, and empower users to access their own data and/or revoke consent. The article by Schaub et al. (2017), which focused on designing effective privacy notices, also implies that the design should be centred on users' needs and characteristics. In their article, they advise that data sharing options should be understandable and easy to use. An eHealth service needs indeed to be *easy to use*. This is defined as the belief of a person that “using a particular system would be free of effort.” (Davis, 1989, p. 320). This means that data sharing options designs should be intuitive for users so that they do not meet difficulties when using the technology. Ease of use of the eHealth service is also a factor which, in literature, is usually associated with trust and privacy, as ease of use can positively influence low privacy concerns and trust (Featherman et al., 2010).

Based on this theoretical background, and the necessity to develop actionable interface and interaction design guidelines for creating health data sharing options, we conducted an experimental design study. We tested two different approaches towards data sharing options. The aim was to find an answer to the question of which of the two designs was the best in terms of trust, privacy concerns, information control and ease of use. The results could help interface and interaction designers to create designs for data sharing options that can enhance the experience of the user. To do that, we created six hypotheses. The first hypothesis focuses on the differences between the two approaches.

H1 There is a significant difference between the two data sharing options designs in terms of ease of use, trust, privacy concerns, and information control.

The remaining hypotheses were explorative. The aim was to find how ease of use, privacy concerns, information control and trust make up the experience the user has when interacting with data sharing options notices. Following the literature, discussed in the Background section, six hypotheses were formulated:

H2 Trust and privacy concerns are negatively correlated.

H3 Ease of use of the design positively influences trust.

H4 Ease of use negatively influences privacy concerns.

H5 Privacy concerns negatively influence information control.

H6 Information control negatively influences privacy concerns.

## 2 Method

To test the hypotheses, an online design experiment with a between-subjects design was used.

### 2.1 Study context

**Fig. 1:**
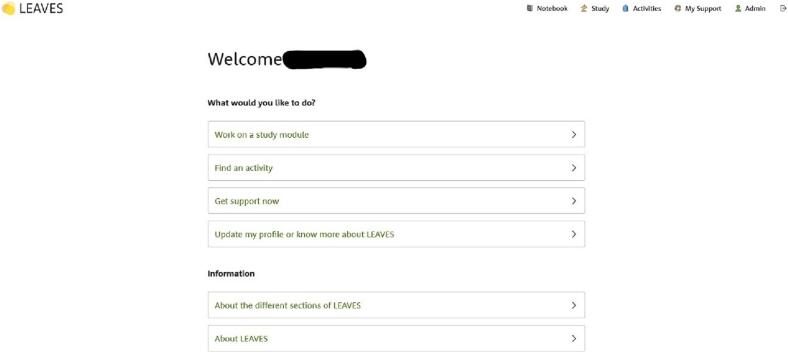
Homepage of LEAVES.

This study has been conducted within the development process of LEAVES (van Velsen et al., 2020). LEAVES is a self-help eMental health service for older adults that have lost their spouse. It offers a human centred design intervention (based on the LIVIA program (Brodbeck et al., 2019)) that supports older adults in their mourning process and helps them to build a new life without their loved one. Fig. 1 shows the homepage of LEAVES. Here users can decide, for example, if working on a study module, finding an activity, or getting support immediately.

During the use of LEAVES, different types of personal health data are stored (for instance, demographics, information about the passing of the spouse, mental health parameters, data end-users enter as part of the therapy, and usage). It is important that some of this data are shared (or not) with different parties. For instance, mental health parameters could be used by the user's doctor to monitor or check the health state of the patient. Accordingly, LEAVES need to have data sharing options to enable users to choose what they want to share and with whom.

### 2.2 Material

Participants were introduced to one of the two approaches via a written scenario and a screenshot, depicting the approach in terms of interface and interaction design. The scenario was created to let participants identify with the envisioned end-user of LEAVES, via the persona of Monika. Monika is a 72 year old widow who is struggling with the death of her partner and, accordingly, decides to use LEAVES. During the onboarding process, she needs to understand and decide which data she wants to share and with whom.

**Fig. 2:**
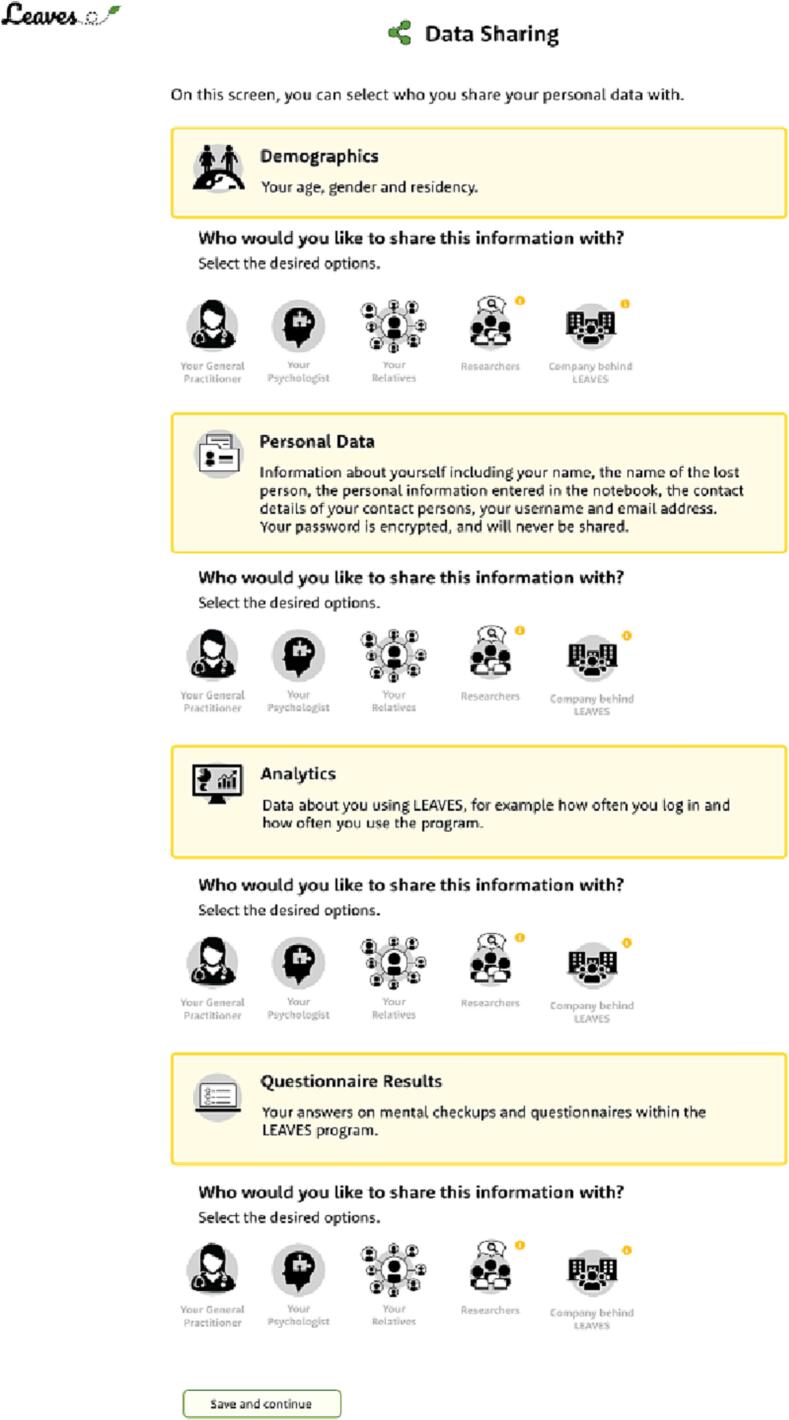
Data sharing design of LEAVES – data perspective.

**Fig. 3:**
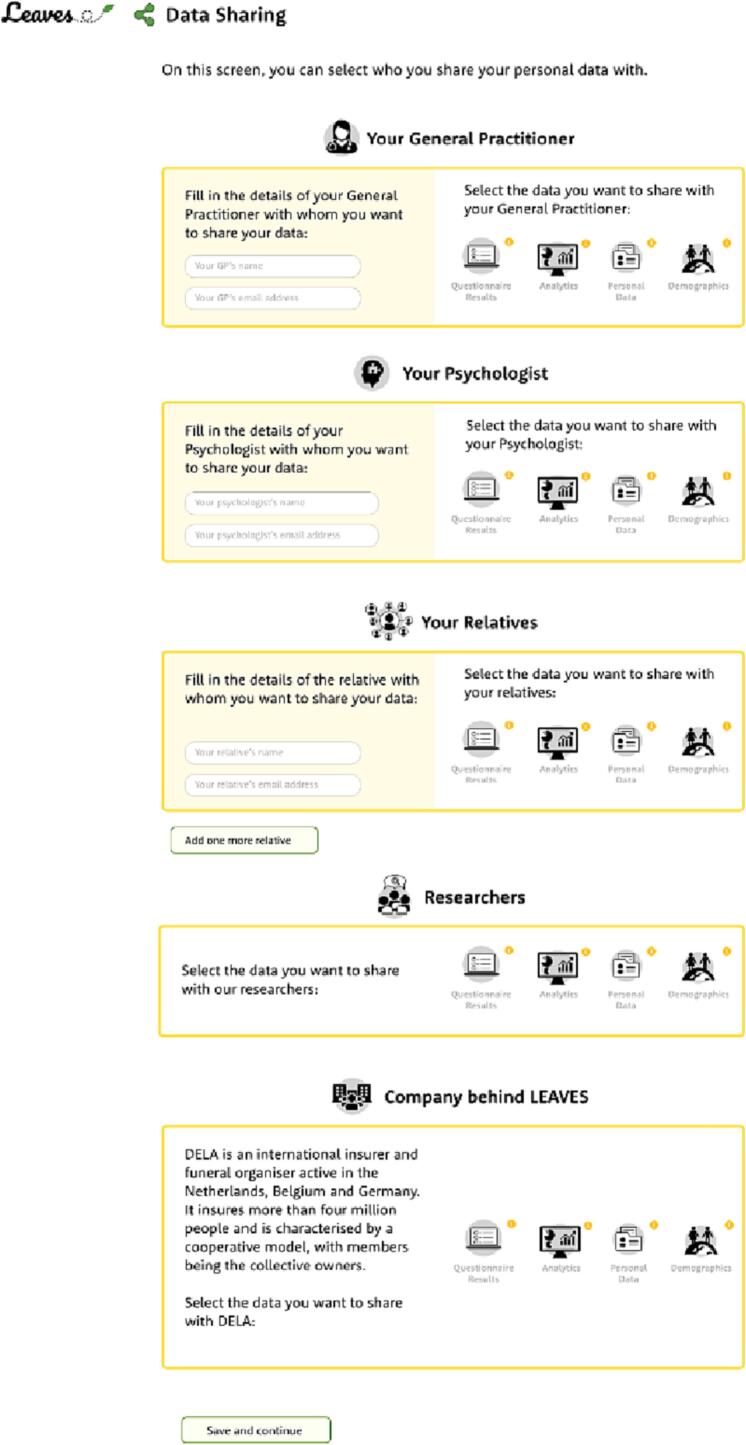
Data sharing design of LEAVES – party perspective.

In the first approach and design (see Fig. 2), data was the focal point – data perspective. In the yellow rectangles, the different types of data that are collected are showed on an abstract level with several, more detailed examples (demographics, personal data, analytics, and questionnaire results). For each type of data, the end-user can specify with whom the service is allowed to share this data (the General Practitioner, psychologist, relatives, researchers, and/or the company behind LEAVES). Above some of the parties there is a small circle with a ‘i’. By clicking on it, users can get more information on the way the data will be used by that particular party. In the second approach and design (see Fig. 3), the actor or organization to share data with was the focal point – party perspective. Per each party, there is a rectangle with a yellow border where users can fill in or find information about the party. Additionally, users can select which data they want to share. Above the data types there is a small circle with a ‘i’. By clicking on it, users can get more information on the data that can be shared. The sharing options designs were based on our knowledge of the LEAVES service and the results from previous usability tests that we performed during the project. Accordingly, users' needs and characteristics were taken into consideration by making these designs user-centred, as it is also advised by Cavoukian (2009), Jensen and Potts (2007), and Schaub et al. (2017).

### 2.3 Measure

At the beginning of the study, participants were asked to provide their gender, age, and educational level. This to identify possible associations with the main variables. A questionnaire with items validated in previous studies was created to measure perceived ease of use, perceived privacy concerns, perceived trust, and perceived information control (Appendix A). Agreement with all statements was assessed on a 5-point Likert scale, ranging from ‘1 = strongly disagree’ to ‘5 = strongly agree’. Finally, via an open question, participants could state if they had any further remarks regarding the design they had seen.

### 2.4 Participants recruitment

The link to the questionnaire with a small description of the study was posted on social media channels of Roessingh Research and Development (RRD), the research centre in which this research was conducted, and it was sent to the participant panel of RRD, mostly including elderly people. Finally, other participants were reached through the snowball method, thus by asking people to share the link with acquaintances. Even if we preferred to have elderly people as our target group, we decided that the minimum age to participate had to be 18 years old. This is to include all possible generations which later will also be using eHealth technology like LEAVES and to explore if there was a difference in the results when analysing the data for age. Finally, we focused on Western Europe participants, as the LEAVES project is a European project.

### 2.5 Analyses of data

After recoding the items which had a negative connotation, precisely the ones for privacy concerns and the second and fourth ones for trust, the variables were formed and the reliability of the construct was measured. To measure the difference between the two data sharing options designs, four *t*-tests were conducted. Afterwards, correlation analysis was conducted. Backward stepwise linear regression analyses were conducted to explore the coming about of the dependent variables ease of use, privacy concerns, trust, and information control.

### 2.6 Ethics

Once participants had opened the link, they were given information about the study and data usage. Additionally, they were given the right to withdraw from the study whenever they wanted. By going on with the study, they consented to use the information given for research purposes. The nature of this internet-based survey among healthy volunteers from the general population does not require formal medical ethical approval according to Dutch law.

## 3 Results

A total of 123 responses were received. For the *t*-test, we compared the designs per variable and used both complete and incomplete responses. For the correlation and regression analyses, only considered the 100 complete responses.

In the first condition (*N* = 66), 64 % of the participants were women and 36 % were men. Additionally, 5 % had lower education, 29 % had secondary education, and 66 % had high education. The mean for age was 58.86 (*SD* = 19.57) with people ranging from 19 to 82 years old (a 0 as outlier). The mode was 73 years old and the median 66 years old. Table 1 shows the descriptive statistics of the data for the first condition.

**Table 1:** Descriptive statistics first condition.

	*N*	Mean	SD	Min	Max
Gender	66	1.64	0.49	1	2
Age	65	58.86	19.57	0	82
Education	66	2.62	0.58	1	3
Ease of use	66	3.54	0.90	1	5
Privacy concerns	60	3.36	0.69	1.43	5
Trust	55	3.31	0.57	2	4.60
Information control	55	3.53	0.83	1	5

In the second condition (*N* = 57), 49 % of the participants were women, 49 % were men, and 2 % selected ‘other’. Additionally, 2 % had lower education, 18 % had secondary education, and 80 % had high education. The mean for age was 54.25 (*SD* = 21.36) with people ranging from 23 to 84 years old (a 0 as outlier). The mode was 72 years old and the median 62 years old. Table 2 shows descriptive statistics of the data for the second condition.

**Table 2:** Descriptive statistics second condition.

	*N*	Mean	SD	Min	Max
Gender	57	1.53	0.54	1	3
Age	57	54.25	21.36	0	84
Education	57	2.79	0.45	1	3
Ease of use	57	3.47	0.86	1	5
Privacy concerns	50	3.12	0.80	1.57	5
Trust	45	3.12	0.77	1.20	4.60
Information control	45	3.22	0.79	1	5

### 3.1 Reliability of measurement constructs

The reliability of all construct was measured. This was met as all values were higher than 0.70 (see Table 3).

**Table 3:** Reliability of constructs.

Variable	Cronbach's alpha
Ease of use	0.89
Privacy concerns	0.85
Trust	0.78
Information control	0.81

### 3.2 Differences between designs

Four independent samples *t*-tests were conducted to test H1 that there is a difference between the two data sharing options designs in terms of ease of use, trust, privacy concerns, and information control. There was no significant effect for ease of use (*t*(121) = 0.42, *p* = .68). The same test found no significant effect for privacy concerns (*t*(108) = 1.74, *p* = .08) or trust (*t*(98) = 1.47, *p* = .14).

For information control, a significant difference (*t* (97) = 2.25, *p* = .03) was found. People in the data perspective condition (*M* = 3.57, *SD* = 0.76) gave higher scores for information control than people in the party perspective condition (*M* = 3.22, *SD* = 0.79). This means that H1 was only met for the factor of information control.

### 3.3 Exploring the correlations between variables

Correlations between the different factors were assessed. As can be seen in Table 4, trust and privacy concerns are significantly positively correlated. When there is trust in the eHealth service there are less privacy concerns. Therefore, H2 was met.

**Table 4:** Correlation analysis.

	Ease of use	Privacy concerns	Trust	Information control	Age	Gender
Ease of use						
Privacy concerns	0.21⁎					
Trust	0.47⁎⁎	0.47⁎⁎				
Information control	0.38⁎⁎	0.18	0.43⁎⁎			
Age	−0.24⁎	−0.15	−0.16	0.01		
Gender	0.06	−0.04	0.09	0.04	−0.33⁎⁎	
Educational level	−0.04	−0.10	−0.10	−0.23⁎	−0.06	−0.12

⁎ Correlation is significant at the .01 level (2-tailed).

⁎⁎ Correlation is significant at the .001 level (2-tailed).

Because some correlations between variables were found and we wanted to explore the data, we decided to conduct backward stepwise linear regression analyses. First, a backward stepwise linear regression was used to explore the influence on ease of use of the following variables: age, gender, educational level, privacy concerns, trust, and information control. At each step, variables were chosen based on *p*-values. In Table 5, it is shown that trust, information control and age were upheld as significant predictors that in combination contributed to ease of use, *F* (3, 96) = 13.54, *p* < .001, with and *R*^2^ of 0.30. A possible relevant result in this analysis could be the influence of age on ease of use of which the correlation was already found. This analysis shows that, in this model, being older negatively influences ease of use, *b* = −0.01, *t* (96) = −2.17, *p* = .03.

**Table 5:** Backward regression analysis with ease of use as dependent variable.

	*b*	*SE*	*β*	*p*
(Constant)	1.68	0.47		<.001
Trust	0.44	0.12	0.34	<.001
Information control	0.25	0.10	0.23	.02
Age	−0.01	0.004	−0.19	.03

Second, a backward stepwise linear regression was used to explore the influence on privacy concerns of the following variables: age, gender, educational level, ease of use, trust, and information control. At each step, variables were chosen based on *p*-values. In Table 6, it is shown that trust was upheld as significant predictor that contributed to privacy concerns, *F* (1, 97) = 27.19, *p* < .001, with and *R*^2^ of 0.22. This means that H4, H6 were not met because neither ease of use nor information control influence privacy concerns.

**Table 6:** Backward regression analysis with privacy concerns as dependent variable.

	*b*	*SE*	*β*	*p*
(Constant)	1.53	0.33		<.001
Trust	0.53	0.10	0.47	<.001

Third, a backward stepwise linear regression was used to explore the influence on trust of the following variables: age, gender, educational level, ease of use, privacy concerns, and information control. At each step, variables were chosen based on *p*-values. In Table 7, it is shown that ease of use, privacy concerns, and information control were upheld as significant predictors that in combination contributed to trust, *F* (3, 95) = 23.07, *p* < .001, with and *R*^2^ of 0.42. The H3 that ease of use positively influences trust was met, *b* = 0.23, *t* (95) = 3.57, *p* < .001.

**Table 7:** Backward regression analysis with trust as dependent variable.

	*b*	*SE*	*β*	*P*
(Constant)	0.68	0.31		.03
Ease of use	0.23	0.07	0.30	<.001
Privacy concerns	0.32	0.07	0.36	<.001
Information control	0.20	0.07	0.25	.004

Finally, a backward stepwise linear regression was used to explore the influence on information control of the following variables: age, gender, educational level, ease of use, privacy concerns, and trust. At each step, variables were chosen based on p-values. In Table 8, it is shown that ease of use, trust, and educational level were upheld as significant predictors that in combination contributed to information control, *F* (3, 95) = 11.15, *p* < .001, with and *R*^2^ of 0.26. As privacy concerns do not significantly influences information control, H5 was not met.

**Table 8:** Backward regression analysis with information control as dependent variable.

	*b*	*SE*	*β*	*P*
(Constant)	0.2.27	0.57		<.001
Ease of use	0.22	0.09	0.23	.02
Trust	0.37	0.12	0.30	.004
Educational level	−0.31	0.14	−0.19	.03

At the end of the questionnaire, participants could write down feedback or comments. We received 16 replies in both condition 1 and condition 2. Several participants commented on the designs themselves. A participant in condition 1 mentioned that they would have also wanted to have the possibility to select that they do not want to share the data with anyone. Related to this, a participant in condition 2 said that it is not explicitly stated that you can choose to not share some data. A few participants were concerned about the lack of information regarding the kind of data that could be shared and with whom these data can be shared. Additionally, it was not clear how this data would be used. According to a participant, it would have been nice to have more concrete examples of the situations data are important to share.

Other participants gave comments on the trust they have in the system. One participant from condition 2 was positive about the design of LEAVES in terms of trust by saying that it makes you confident that you can trust it. Moreover, another participant in condition 1 commented it seemed that the LEAVES program clearly informed about the choices a user might have, but that there can always be something going wrong when sharing personal data on the internet.

These comments can be seen as positive in terms of trust. Nonetheless, this and other participants were genuinely concerned about the spread of personal information on the internet. Finally, another participant was worried about the data stored by different stakeholders as the way in which this data was protected was not explained. Linked to this, a participant advised adding a disclaimer at the beginning stating that the personal data are secured and exclusively shared with the people the user decides.

## 4 Discussion

In this study, we compared two different approaches towards data sharing options designs: a data perspective and a party perspective. We did this by exploring the concepts of ease of use, privacy concerns, information control, and trust. Both data sharing options designs were based on the user centred design approach and designing for privacy. This might explain why both designs scored quite high on ease of use, (no) privacy concerns, trust, and information control. Differences in appreciation between the two different approaches were limited to one factor: information control. Control was higher in the data perspective condition where the data of a user were given more importance than the party they share the data with. Following the subprinciple ‘Appropriate defaults’ of the STRAP framework (Jensen and Potts, 2007, pp. 50–51), putting the data at the centre is indeed reflecting the biggest concerns for users.

From the results, we could say that trust in technology is a core factor in designing sharing options. A system that is easy to use, is designed for privacy, and it makes users feel that they have control of their information is a system to trust and vice versa. That trust is a fundamental factor to reduce privacy concerns and, in turn, to increase users' willingness to share data in eHealth is highlighted in the paper by Arfi et al. (2021). The authors explain how in an eHealth service where data need to be shared, privacy concerns can decrease the trust a patient has in the service and the willingness to share data. This was also found in the study by Belfrage et al. (2022).

Privacy concerns are indeed a big issue in data sharing options. Following the literature, a service should be easy to use and should give feelings of control over information. Otherwise, users' privacy perceptions might be negatively influenced (Featherman et al., 2010). In this study, however, these hypotheses were not met. Nonetheless, from correlation analysis, it can be said that if a design is easy to use, users also have fewer privacy concerns and vice versa. Having privacy concerns did not influence the control that a person has over his or her data, and no correlation was found between these two variables. Nonetheless, in literature, it was found that when users have privacy concerns, they will be less willing to share their data, as they are afraid to lose control (Abdelhamid et al., 2017). A reference to control can also be found in the definition of privacy, which is “the ability of an individual to exercise control over their personal data held by others.” (Sahama et al., 2013, p. 250). As, in our study, trust was positively correlated with lower privacy concerns and information control, and it is influenced by both of them, it might be the case that there is an indirect association between information control and privacy concerns.

Much research done on trust, acceptance and intention to use technology, investigates the influence of trust and ease of use. In some of these studies, ease of use was also found to positively influence trust (Liébana-Cabanillas et al., 2014; Corritore et al., 2012). Additionally, the correlation between these two variables was also highlighted in McKnight et al. (2002). This underlines that ease of use and trust are associated and that a design of an eHealth service needs to be easy to use to enhance trust.

From the comments of participants, the first design could still be improved and this can also be done by following guidelines on how to design for privacy. First, users might need to have more explanation on how the data will be used and the purpose to collect those data, also to prevent people from thinking that their data can be used for commercial goals. Consequently, by following the subprinciples ‘Presented in context’ and ‘Appropriate defaults’ (Jensen and Potts, 2007, pp. 50–51), users' feeling of privacy might benefit from information about the way data are used by each stakeholder and why they are used. At the beginning of the data sharing options, it should be stated clearly that the data are secured and exclusively shared with the people that are selected by the user. This will increase feelings of integrity and security towards the system (Jensen and Potts, 2007). Additionally, there should be explicitly written that users do not have to give permission to share some data with someone if they do not want to. This is also according to the ‘Choice and Consent’ principle of the STRAP framework (Jensen and Potts, 2007, pp. 50–51). In line with this, according to the ‘Available, Accessible, and Clear’ subprinciple (Jensen and Potts, 2007, pp. 50–51) and Cavoukian (2009), users should be said that they can change their sharing options whenever they want and where they can do that. By applying these recommendations in data sharing options design, trust and feelings of privacy and information control in the intervention could be met.

### 4.1 Limitations and strengths

This study has some limitations. Due to the study design, participants did not have the possibility to see both designs and compare them. Only seeing the designs was probably not enough to understand what the program LEAVES is about or who was providing it. This might have made it more difficult for participants to answer the questions about trust in the service. Additionally, participants only saw a screenshot and this might have not given them the possibility to really experience the design and answer the questions more accurately.

This paper also has strengths. We had the possibility to explore designing for privacy (via a quantitative and qualitative approach) by using the data sharing options designs of an existing eHealth technological intervention – LEAVES, which was already based on users' inputs and characteristics. This allows us to better explore the data and have a base to find important factors in designing for privacy, like, for example, information control. Moreover, the focus of studies that investigate designing for privacy is usually on how the system ensures that the data are stored properly and according to regulations, for example with encryption. This paper, however, provides guidelines for interface and interaction design that can function as the front-end of these architectural decisions.

### 4.2 Concluding remarks

In this study, we took a design perspective in health data sharing. Assuming that health data sharing has good intentions, end-users are served best by letting them control their health data from a data perspective. Additionally, end-users should be given an overview of different types of personal data that are collected, and then let them decide with whom they would like to share this data. Although this approach does not provide benefits for the total experience of the user (e.g., ease of use, trust), it does give high feelings of information control. In order to generate trust in data sharing functionality, the complete user experience does need to be positive. For that, design for privacy recommendations were also provided.

## Declaration of competing interest

The authors declare that they have no known competing financial interests or personal relationships that could have appeared to influence the work reported in this paper.

## Figures and Tables

**Table:** 

Variable	Items	Source
Ease of use	• In this page of the LEAVES program everything is easy to understand • This page of the LEAVES program is simple to use, even when using it for the first time • It is easy to find the information I need from this page of the LEAVES program	Belanche et al. (2012)
Privacy concerns	The information submitted on the LEAVES program… • … can be used in a way I did not foresee • … can be used against you by someone • … can become available to someone without your knowledge • … can become available to someone you do not want to (e.g. children, doctors, therapists, etc.) • … can be misinterpreted • … can be continuously spied on (by someone unintended) • … can be used for commercial purposes (e.g. market research, advertising)	Krasnova et al. (2010)
Trust in an eHealth service	• I can trust that possible problems with the LEAVES program will be solved properly • I can trust the LEAVES program less than other online services, such as Bol.com and the website of my municipality • I feel at ease when working with the LEAVES program • I do not like to enter my personal data on the LEAVES program	van Velsen et al. (2017)
Information control	• I was informed about the personal information the LEAVES program would collect about me • The LEAVES program explained why personal information was being collected • The LEAVES program explained how personal information collected about me would be used • This website gave me a clear choice before using personal information about me	Taylor et al. (2009)
